# Arhovin, a New Internal Antigonorrheic

**Published:** 1906-05

**Authors:** 


### ABSTRACTS.

### Arhovin, a New Internal Antigonorrheic.

F. Burchard and A. Schlockow, Berlin, Germany, (*Die Medicinische Wochen*. November 30, 1903) say that arhovin, an addition-product of diphenylamine and esterified thymylbenzoic acid, was subjected by us to extensive physiological and therapeutic experimentation. It is a fluid of aromatic odor and cool, slightly burning taste, with a boiling point of 218° C., almost insoluble in water, but readily soluble in alcohol, ether and chloroform. Its gastric absorption is exceedingly rapid (15 minutes). The exact form in which it is excreted is as yet undetermined; but the dark green action of the urine with 1% ferric chloride solution points to phenyl-hippuric acid, the thymol being changed into thymol-glycuronic acid.

In our entire series of experiments on healthy persons and patients there was never the slightest indication of any undesirable effect on the organism. On the contrary, the condition of the subjects invariably improved, even when the remedy was given continuously for weeks. The dose was four grains five times daily in capsules. By mouth it is best given in capsule form.

In some persons the urine became clear and absolutely odorless at the beginning; in others it retained normal color and bouillon-like smell. Cloudy urine was quickly cleared; but arhovin had no influence on phosphaturia. The urine was rendered powerfully bactericide and entirely inhibited growth in pure cultures of gonococci, streptococci, and the like.

Urinary reaction in some cases became strongly acid; in others it remained unchanged. Usually, however, acidity was permanently increased, preventing or arresting ammonical decomposition. It seems that the acid and bactericide urine renders the vesical and urethral mucosa unsuitable for bacterial growth.

Arhovin is a prophylactic of gonorrheal joint inflammation and of endocarditis. Most if not all of these affections are metastatic; the direct cause of the disease is brought to the joint by the blood current and finds a nidus in the synovial membrane. Injuries and exposures to cold are but predisposing agents, if cognised as antiseptics; but their toxicity has stood in the way in the blood, but appear therein or on pus cells and also on particles or solid uric acid. The advent of the latter alone in the synovial sacs may cause swelling and inflammation of the joints. Since arhovin changes the fairly insoluble uric acid into the very soluble hippuric acid, it may well prevent the deposition of the crystals in the joints.

Diphenylamine, thymol and benzoic acid have long been recognised as antiseptics; but their toxicity have stood in the way of their internal use. We regard arhovin, whose absolute nonpoisonousness we have proved by months of experimentation, as a most fortunate combination.

With regard to the simultaneous use of the customary injections, individualism is desirable, though we do not believe that a drug capable of destroying the gonococci and of sterilising the nutrient media within three weeks needs any such support. Arhovin is especially advantageous in female gonorrhea, where there are practical difficulties in the way of local treatment.

Arhovin has no notable effect on the amount of urine excreted. Kidney disease is no contraindication to its use. Microscopic examination for gonococci were made in every case and always gave negative results.

